# Psychoneurological Symptoms and Biomarkers of Stress and Inflammation in Newly Diagnosed Head and Neck Cancer Patients: A Network Analysis

**DOI:** 10.3390/curroncol29100559

**Published:** 2022-09-28

**Authors:** Angelina M. M. Santoso, Femke Jansen, Carel F. W. Peeters, Robert J. Baatenburg de Jong, Ruud H. Brakenhoff, Johannes A. Langendijk, C. René Leemans, Robert P. Takes, Chris H. J. Terhaard, Annemieke van Straten, Irma M. Verdonck-de Leeuw

**Affiliations:** 1 Department of Clinical, Neuro and Developmental Psychology, Faculty of Behavioral and Movement Sciences & Amsterdam Public Health Research Institute, Vrije Universiteit Amsterdam, Van der Boechorststraat 7, 1081 BT Amsterdam, The Netherlands; 2 Cancer Center Amsterdam Research Institute, Amsterdam UMC, Vrije Universiteit Amsterdam, P.O. Box 7057, 1007 MB Amsterdam, The Netherlands; 3 Department of Otolaryngology—Head and Neck Surgery, Amsterdam UMC, Vrije Universiteit Amsterdam, P.O. Box 7057, 1007 MB Amsterdam, The Netherlands; 4 Department of Epidemiology and Data Science, Amsterdam UMC, Vrije Universiteit Amsterdam, P.O. Box 7057, 1007 MB Amsterdam, The Netherlands; 5 Mathematical & Statistical Methods Group (Biometris), Wageningen University & Research, P.O. Box 16, 6700 AA Wageningen, The Netherlands; 6 Department of Otolaryngology and Head and Neck Surgery, Erasmus Cancer Institute, Erasmus MC, 3015 GD Rotterdam, The Netherlands; 7 Department of Radiation Oncology, University of Groningen, University Medical Centre Groningen, 9713 GZ Groningen, The Netherlands; 8 Department of Otorhinolaryngology and Head and Neck Surgery, Radboud University Medical Center, Radboud Institute for Health Sciences, 6525 GA Nijmegen, The Netherlands; 9 Department of Radiotherapy, University Medical Center, 3584 CX Utrecht, The Netherlands

**Keywords:** sleep quality, depression, anxiety, pain, fatigue, biomarker, inflammation, cortisol, head and neck cancer, network analysis

## Abstract

Psychoneurological symptoms are commonly reported by newly diagnosed head and neck cancer (HNC) patients, yet there is limited research on the associations of these symptoms with biomarkers of stress and inflammation. In this article, pre-treatment data of a multi-center cohort of HNC patients were analyzed using a network analysis to examine connections between symptoms (poor sleep quality, anxiety, depression, fatigue, and oral pain), biomarkers of stress (diurnal cortisol slope), inflammation markers (c-reactive protein [CRP], interleukin [IL]-6, IL-10, and tumor necrosis factor alpha [TNF-α]), and covariates (age and body mass index [BMI]). Three centrality indices were calculated: degree (number of connections), closeness (proximity of a variable to other variables), and betweenness (based on the number of times a variable is located on the shortest path between any pair of other variables). In a sample of 264 patients, poor sleep quality and fatigue had the highest degree index; fatigue and CRP had the highest closeness index; and IL-6 had the highest betweenness index. The model yielded two clusters: a symptoms—cortisol slope—CRP cluster and a IL-6—IL-10—TNF-α—age—BMI cluster. Both clusters were connected most prominently via IL-6. Our findings provide evidence that poor sleep quality, fatigue, CRP, and IL-6 play an important role in the interconnections between psychoneurological symptoms and biomarkers of stress and inflammation in newly diagnosed HNC patients.

## 1. Introduction

Head and neck cancers (HNC) include a range of cancers located in the nasal cavity, paranasal sinuses, oral cavity, pharynx, larynx, and salivary glands. Every year, more than 550,000 people in the world are newly diagnosed with HNC [1]. The period between diagnosis of HNC and the start of treatment is often stressful. Patients usually present to the healthcare facilities with orofacial pain, swallowing problems, neck lump, hoarseness, or weight loss [2]. Besides dealing with cancer symptoms and multiple diagnostic procedures, HNC patients are often anxious about the disease prognosis and effect of cancer treatment on their daily life [3]. Newly diagnosed HNC patients often report not only pain (especially in the mouth and neck area) [4] but also poor sleep quality [5], psychological distress [6], and fatigue [7]. These symptoms often co-occur in cancer patients, and they are often referred to as ‘psychoneurological symptoms’ [8,9].

It is hypothesized that psychoneurological symptoms are related to biomarkers of stress and inflammation. Cortisol, a stress-related hormone produced in the hypothalamic-pituitary-adrenal (HPA) axis, plays an important role in the regulation of metabolism, the cardiovascular system, and the inflammation response [10]. Under normal circumstances, the concentration of cortisol follows a diurnal rhythm: it is high at wakening, increases rapidly until it peaks within 30–45 min after awakening, and then decreases steadily towards its lowest level around midnight [11]. This normal fluctuation is found to be altered upon stress, e.g., in the presence of psychoneurological symptoms: the cortisol peak either shifts to a later timepoint or completely disappears, showing a blunted or flatter diurnal cortisol slope [12]. Such diurnal rhythm disruption (i.e., flatter diurnal cortisol slope) was found to be associated with higher inflammatory markers [13]. Higher inflammation is hypothesized to promote HNC progression by stimulating cancer proliferation, migration, and angiogenesis [14].

Previous studies in newly diagnosed lung, ovarian, and colorectal cancer patients suggested associations between: (1) worse sleep quality and a flatter cortisol slope [15] and (2) worse psychoneurological symptoms (i.e., poor sleep quality, anxiety, depression, and fatigue) and a higher level of inflammation markers [16,17,18]. In newly diagnosed HNC patients specifically, significant associations were demonstrated between fatigue and higher levels of the inflammation markers interleukin-6 (IL-6) and c-reactive protein (CRP) [19], and between pain and a higher level of CRP [20]. To the best of our knowledge, no study has investigated associations between psychoneurological symptoms, cortisol, and inflammatory markers altogether in newly diagnosed HNC patients. This information is needed to understand the pathophysiology of psychoneurological symptoms in newly diagnosed HNC patients and, eventually, to design a better strategy for monitoring and intervention of these symptoms.

Psychoneurological symptoms and biomarkers of stress and inflammation may influence each other by providing positive or negative feedback [21,22]. Hypothetically, the association between two specific elements (i.e., a specific symptom or a biomarker) may also be influenced by their associations with the other remaining elements. Network analysis takes this complexity into account, and it is used increasingly in mental health research [23]. Therefore, we used this novel approach to examine the associations between psychoneurological symptoms (poor sleep quality, anxiety, depression, fatigue, and oral pain), and biomarkers of stress (a diurnal cortisol slope) and inflammation (CRP, IL-6, interleukin-10 (IL-10), and tumor necrosis factor alpha (TNF-α)) among newly diagnosed HNC patients. Based on the available literature, we expected positive connections between psychoneurological symptoms and inflammation markers (i.e., worse symptoms are associated with higher inflammation markers) [16,17,18,19,20], and negative connections between the cortisol slope with psychoneurological symptoms and inflammation markers (i.e., a flatter cortisol slope is associated with worse psychological symptoms and with higher inflammation markers) [13,15].

## 2. Patients and Methods

### 2.1. Study Population

Baseline data from the ongoing NETherlands Quality of Life and Biomedical Cohort study in head and neck cancer (NET-QUBIC) were used [24]. From March 2014 to June 2018, all HNC patients who were newly diagnosed at 5 university hospitals and 3 satellite hospitals were assessed for their eligibility to participate in the study. The study was approved by the Medical Ethical Committee of the VU University Medical Center Amsterdam (2013.301(A2018.307)-NL45051.029.13).

Descriptive characteristics were obtained as follows. Sex, age, and clinical characteristics (HNC subsite, HNC stage, performance status, and comorbidity) were obtained from electronic medical records. Education level and living situation were obtained from interviews or questionnaires. Smoking status (smoking daily at baseline) and excessive alcohol consumption (>14 units of alcohol per week for women or >21 units of alcohol per week for men) were self-reported using a study-specific questionnaire. BMI was calculated based on height and weight (weight (kg)/height (m)), which were measured using a standardized procedure during a home visit. Among these characteristics, two continuous variables were included in the network model as covariates as they were associated with higher inflammatory markers, namely, age [25] and BMI [26].

### 2.2. Inclusion and Exclusion Criteria of NET-QUBIC Study

Inclusion criteria were: 18 years of age or older; diagnosis of squamous cell carcinoma of the oral cavity, oropharynx, hypopharynx, or larynx, or neck lymph node metastasis of an unknown primary tumor; intention of curative treatment; and the ability to write, read, and speak Dutch. Exclusion criteria included severe psychiatric comorbidity (e.g., schizophrenia, Korsakoff’s syndrome, severe dementia), as participation in the study was thought to be too burdensome. In addition, patients with less prevalent types of HNC (i.e., lymphoma, thyroid cancer, nasopharyngeal cancer, malignancy of skin, or malignancy of salivary glands) were excluded from the study.

### 2.3. Measures

NET-QUBIC encompasses measurements at baseline (before start of treatment) and at 3, 6, 12, 24, 36, 48, and 60 months of follow-up. In this study, we used only the baseline measurements.

Sleep quality was measured using the Pittsburgh sleep quality index (PSQI) [27]. Its validity and reliability have been confirmed in cancer patients [28]. PSQI covers seven domains of sleep quality and disturbances [27], with each domain score ranging from 0 to 3. The PSQI total score ranges from 0 to 21; a higher score indicates poorer sleep quality.

Psychological distress, i.e., depression and anxiety symptoms, was assessed using the 14-item hospital anxiety and depression scale (HADS) [29]. The anxiety (HADS-A) and depression (HADS-D) subscales both consist of 7 items, each ranging from 0 to 3. The sum score of each subscale ranges from 0 to 21; a higher score indicates a higher extent of depression or anxiety symptoms. The HADS was demonstrated to have good psychometric properties in measuring anxiety and depression among cancer patients [30].

Pain was measured using the oral pain subscale of the European organization for the research and treatment of cancer quality of life questionnaire, HNC specific module (EORTC QLQ-H&N35) [31]. This subscale consists of 4 items about whether the patient has had pain in the mouth, jaw, or throat, or soreness in the mouth (further referred to as oral pain). The subscale score ranges from 0 to100; a higher score indicates a worse extent of oral pain [32].

Fatigue was measured using the general fatigue scale of the Multidimensional Fatigue Inventory (MFI-20) [33]. This scale consists of four items, wherein each item is scored on a 5-point Likert scale, yielding a summary score that ranges from 4 (least fatigued) to 20 (most fatigued). This scale has good psychometric properties in measuring fatigue among cancer patients [33].

Saliva collection and cortisol measurement were performed as follows. Patients were instructed to collect saliva at four time points: at awakening, 30 min after awakening, 60 min after awakening, and at 22:00. Patients were given 4 salivette tubes (one for each saliva sample) and were instructed to note the exact time of saliva collection. All samples were collected at home by the patients after a thorough explanation by a trained fieldworker (along with a written guideline) and sent by mail to the coordinating research center (Amsterdam UMC, location VUmc). Upon receipt, the samples were immediately centrifuged, transferred to storage vials, and stored at −20 °C. Saliva cortisol concentrations were measured at the Endocrine Laboratory of the Amsterdam UMC, location AMC, with the use of an isotope-dilution LC-MS/MS method. In short, the internal standard (13C3-labeled cortisol, Isosciences) was added to the samples. Samples were extracted by supported liquid extraction (Biotage) and analysed on a LC-MS/MS (Xevo TQ-S Micro LC-MS-MS System, Waters Corporation, Milford, MA, USA). The lower limit of quantitation was 1.0 nmol/L. The intra-assay variation was 5% and 3% at the cortisol concentrations of 2 and 15 nmol/L, respectively. The inter-assay variation was <9% over the whole concentration range. The diurnal cortisol slope was calculated by substracting the cortisol level at 22:00 from the cortisol level at awakening, divided by the duration (in hours) between these two time-points [34]. A higher value of slope means a steeper decline of cortisol, a lower value of slope means a slower decline, and a negative slope indicates an increasing cortisol level during the day. 

Inflammatory markers (CRP, IL-6, IL-10, and TNF-α) were measured from venous blood samples collected by a research nurse or trained fieldworker. For CRP, blood was collected in a heparine-gel tube. CRP was immediately determined in the laboratories of the participating hospitals according to standard laboratory protocol. For cytokines IL-6, IL-10, and TNF-α, blood was collected in a tube for serum collection with a clot activator. The serum samples were preprocessed according to the standard laboratory protocol and stored at the NET-QUBIC biobank (AmsterdamUMC, location VUmc) at −80 °C until the day of the assay. Cytokines were measured by the laboratory of Clinical Chemistry at AmsterdamUMC, location VUmc, in 500 µL aliquots. Each sample was analyzed using an ELISA-based technology that uses electrochemiluminescence for detection with Meso Scale Discovery Quickplex SQ 120 Imager (cat. # K15049-Series, Meso Scale Discovery, Rockville, MD, USA). The intraassay variations were 4.7% (IL-6), 4.0% (IL-10), and 3.7% (TNF-α) and the interassay variations were 7.9% (IL-6), 5.5% (IL-10), and 8.0% (TNF-α).

### 2.4. Statistical Analysis

Statistical analyses were performed using IBM SPSS (IBM Corp., Armonk, NY, USA) and R (R Foundation for Statistical Computing, Vienna, Austria).

Sociodemographic factors, clinical characteristics, smoking and alcohol use, psychoneurological symptoms, and biomarkers were compared between patients with complete vs. incomplete data: a chi-square test was used for categorical variables, an unpaired *t*-test for normally distributed continuous variables, and a Mann-Whitney U test for non-normally distributed variable. Statistical significance was defined as *p*-value < 0.05. We adjusted for multiple testing by controlling the false discovery rate (FDR) [35].

An undirected network model was used to examine the connections between poor sleep quality, symptoms of anxiety, symptoms of depression, oral pain, fatigue, biomarkers (cortisol slope, CRP, IL-6, IL-10, and TNF-α), and covariates (age and BMI). Only participants with complete data for these 12 variables were included in the analyses (i.e., using a listwise approach). The network was estimated using R package *Rags2Ridges* version 2.2.3 [36]. First, skewed distributions were normalized by nonparanormal transformations using the *huge.npn* function from the *huge* package. Network extraction was then based on targeted ridge estimation of the partial correlation matrix [37]. The resulting partial correlation matrix was sparsified by thresholding, choosing an absolute value ≥ 0.1 as the cut-off value. The resulting networks were conditional independence graphs. In such graphs, the nodes represent the variables (i.e., symptoms, biomarkers, and covariates), and the connections between node-pairs represent a substantive partial correlation, i.e., an association that cannot be conditioned away by the remaining nodes (given the chosen threshold). The network of conditional associations was visualized using the Fruchterman-Reingold approach, which locates highly associated nodes closer to each other [38]. To gain insight into the structural importance of the nodes in the network, we calculated three centrality indices, namely the “degree” (the number of connections for a node), “closeness” (average proximity of a node to all other nodes), and “betweenness” (based on the number of times a node is located on the shortest path between any pair of the other nodes) [39]. Finally, we examined the clustering of nodes (i.e., variables which are closer to each other) using the edge-betweenness approach, also known as shortest-path betweenness [40].

## 3. Results

### 3.1. Study Population

Given the large scope of the study measurements, at baseline, the newly diagnosed NET-QUBIC participants were given the possibility to participate only in certain components of the assessment (i.e., questionnaires, home-based tests, self-collected saliva, and/or blood collection). In this study, using 12 variables of interest, missing values ranged from one variable (in 141 patients) to 11 variables (in 20 patients). The most missing values were found for cortisol slope (46%), sleep quality (24%), fatigue (24%), BMI (23%), anxiety (19%), and depression (19%). Among all 739 patients included in the NET-QUBIC study, 264 patients (36%) had complete data on all twelve variables of interest and were included in the network analysis.

Table 1 provides an overview of characteristics of patients with complete (*n* = 264) vs. those with incomplete data (*n* = 475). Patients with complete data were older (mean age 65 years (SD = 8.2) vs. 62 years (SD = 10.4), adjusted *p* = 0.007), more often lived together with family or relatives (81.4% vs. 70.1%, adjusted *p* = 0.007), and were less fatigued (median = 9.0 (5.0–13.0) vs. 11.5 (6.0–14.0), adjusted *p* = 0.007). No statistically significant difference was found for clinical characteristics, biomarkers, daily smoking status, and excessive alcohol consumption.

### 3.2. Network Analysis

**Figure 1:**
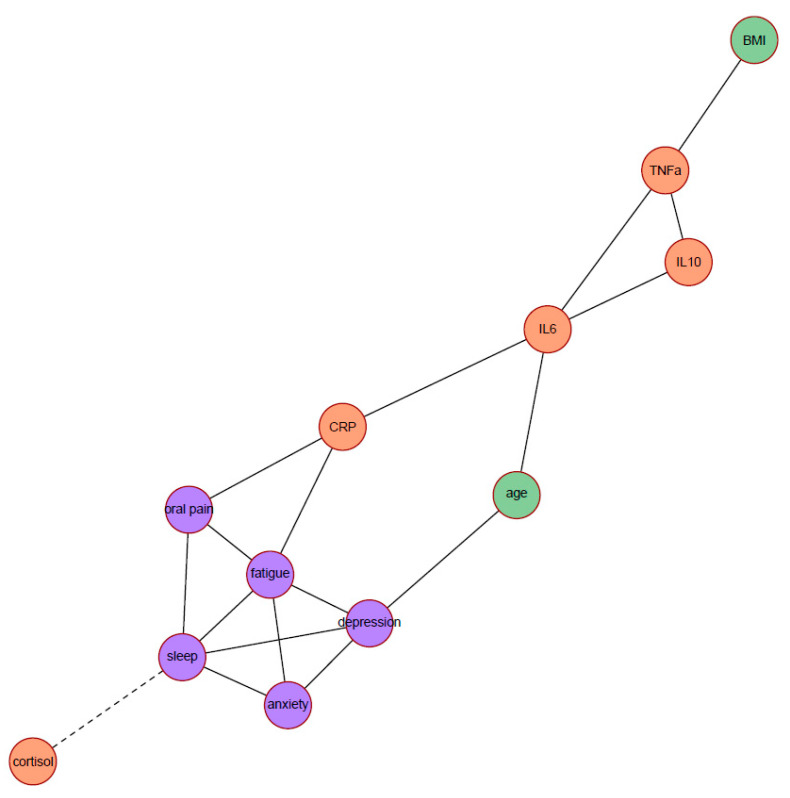
Network displaying the partial correlations between all symptoms (in purple), biomarkers (in salmon orange), and covariates (in green); *N* = 264. Solid edges represent positive partial correlations, dashed edges represent negative partial correlations. The anxiety label indicates the HADS anxiety subscale score; depression, HADS depression subscale score; fatigue, MFI general fatigue score; oral pain, EORTC QLQ-H&N35 oral pain subscale score; sleep, PSQI total score; and cortisol, the diurnal cortisol slope. Abbreviations: BMI, body mass index; CRP, c-reactive protein; IL, interleukin; TNF-α, tumor necrosis factor alpha.

Figure 1 depicts the network of partial correlations between psychoneurological symptoms, biomarkers, and covariates (age and BMI). Solid edges represent positive partial correlations and dashed edges represent negative partial correlations. All edges were positive (indicating that higher levels of symptoms, inflammation markers, or covariates were associated with higher levels of other symptoms, inflammation markers, or covariates), except for the edge between poor sleep quality and the cortisol slope. A higher PSQI total score (poorer sleep quality) was connected with a lower value of cortisol slope (flatter slope).

Centrality indices of all nodes are displayed in Table 2. Based on the three centrality indices (degree, closeness, and betweenness), four nodes had the most important position in the network: fatigue (highest degree and closeness), poor sleep quality (highest degree), CRP (highest closeness), and IL-6 (highest betweenness). Poor sleep quality and fatigue were connected with the most number of nodes (i.e., highest degree): each had 5 connections with other nodes. Fatigue and CRP had the highest closeness index, which means that changes in fatigue or CRP will quickly result in changes of any other node in the network, or vice versa. IL-6 had the highest betweenness, which means that IL-6 is most often an intermediate node on the shortest paths between any pair of nodes. Hence, from the viewpoint of information flow, IL-6 is an important node.

**Figure 2:**
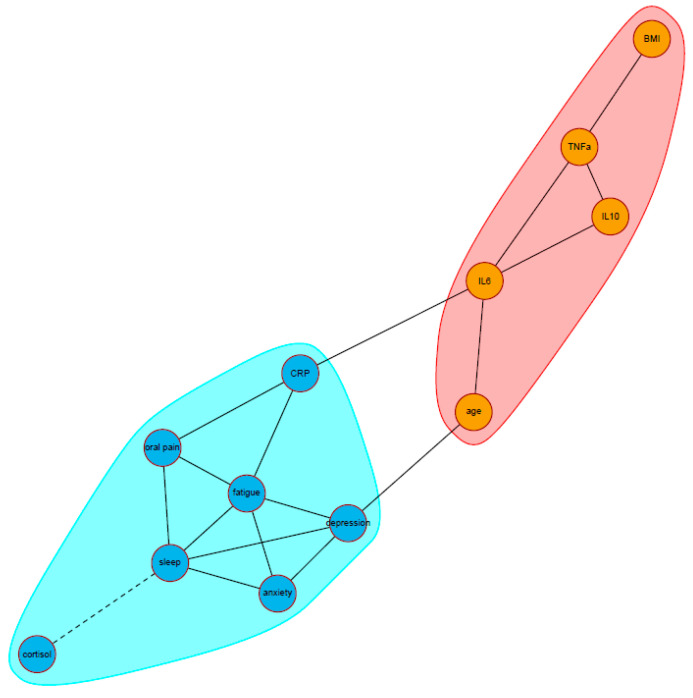
Visualization of the community structure between all symptoms, biomarkers, and covariates, using the edge-betweenness approach (*N* = 264). Solid edges represent positive partial correlations, dashed edges represent negative partial correlations. The two clusters are represented by different colored area (red and blue). The anxiety label indicates a HADS anxiety subscale score; depression, HADS depression subscale score; fatigue, MFI general fatigue score; oral pain, EORTC QLQ-H&N35 oral pain subscale score; sleep, PSQI total score; cortisol, the diurnal cortisol slope. Abbreviations: BMI, body mass index; CRP, c-reactive protein; IL, interleukin; TNF-α, tumor necrosis factor alpha.

Two clusters were identified in the community analysis (Figure 2). The first cluster consisted of all patient-reported psychoneurological symptoms (poor sleep quality, depression, anxiety, fatigue, and oral pain) together with an inflammation marker (CRP) and stress marker (the cortisol slope). The second cluster consisted of the other three inflammation markers (IL-6, IL-10, and TNF-α) and the two covariates (age and BMI). Based on its highest betweenness index, IL-6 was the most important node connecting the two clusters.

## 4. Discussion

To the best of our knowledge, this study was the first to use a network analysis to examine associations between psychoneurological symptoms and biomarkers of stress and inflammation in newly diagnosed HNC patients. All psychoneurological symptoms in our network model were connected with each other as well as with biomarkers in the expected directions: worse symptoms were associated with higher levels of inflammatory markers and a flattened cortisol slope. Two clusters were identified in our network model: all psychoneurological symptoms—cortisol slope—CRP cluster, and the inflammation cytokines (IL-6, IL-10, TNF-a)—age—BMI cluster. Poor sleep quality, fatigue, CRP, and IL-6 were the most central nodes in the network based on the centrality indices.

The central position of poor sleep quality and fatigue among other psychoneurological symptoms suggesting their importance in the network of psychoneurological symptoms and markers of stress and inflammation. Poor sleep quality may worsen fatigue, anxiety, depression, and lower pain tolerance by impairing emotional regulation [41]. As such, newly diagnosed HNC patients often suffer from anxiety, depression, fatigue, and oral pain, which may disrupt their sleep quality [42]. Fatigue has also been demonstrated both as a cause [43] as well as a consequence [44] of psychological distress and pain in cancer patients. Future study using time-series data is needed to gain more insight on the dynamics of these central symptoms in the network model over time.

Based on our network model, IL-6 appeared to be the most important node connecting other non-adjacent nodes, as well as the most important node in terms of the information flow between the two clusters. A previous study hypothesized that IL-6 was produced not only by tumor cells to induce HNC proliferation but also by host cells (e.g., endothelial cells, stromal cells, immune cells) as a response to tumor growth [45]. Furthermore, an accumulative increase of IL-6 induces the release of CRP in a larger quantity, a marker of ongoing systemic inflammation [46]. In accordance with a previous study in newly diagnosed HNC patients [19], we also found that higher CRP levels is also associated with worse pain and fatigue.

In this study, the association between stress biomarkers and psychoneurological symptoms seemed to take place particularly through the connection of a flatter cortisol slope with poor sleep quality. In contrast with earlier findings in general adult population [13], our network model did not indicate a connection between the cortisol slope and inflammatory cytokines. This finding may be explained by a decreased sensitivity of immune cells towards cortisol (i.e., cortisol resistance) upon systemic inflammation [47], which is hypothesized to play a key role in cancer pathogenesis [48]. A study in HNC patients before and 1 month after treatment found that increasing inflammation and fatigue was associated with increasing cortisol resistance over time, but this study did not include poor sleep quality in their analysis [49]. Nonetheless, our findings may suggest that poor sleep quality plays an important role in the association between HPA-axis disruption, higher inflammation, and psychoneurological symptoms through several pathways. First, poor sleep quality and a flattened cortisol slope may aggravate each other. Second, the cortisol-resistant immune cells may sustain their inflammatory state by ignoring anti-inflammatory cues from cortisol. Both prolonged poor sleep quality and sustained inflammation may lead to more fatigue and the eventual worsening of the other psychoneurological symptoms. This hypothesis has yet to be tested.

A strength of this study was that we examined associations between psychoneurological symptoms, cortisol diurnal slope, and inflammation markers by network analysis using a large study cohort of newly diagnosed HNC patients. Our findings also need to be interpreted with caution. First, as we analyzed only patients with complete data, our findings may not be fully representative of the HNC patients that did not have complete data (those who were younger, lived alone, and were more fatigued). Second, we measured inflammatory marker levels in blood at one time point of the day, and these levels may vary throughout the day. However, collecting blood samples multiple times in a day may be too burdensome for the patients. To deal with this issue, future investigations may consider using serial saliva samples to measure inflammatory markers [50]. Third, we only measured IL-6, IL-10, and TNF-α as they were the most frequently studied markers, and we did not measure the concentration of other cytokines. A further study including other inflammatory cytokines is needed for deeper insight on the inflammatory process in the network. Fourth, we did not control for different subgroups of HNC patients, for example, based on HNC location or stage. Future study is needed to examine whether the network of psychoneurological symptoms, the cortisol diurnal slope, and inflammation differ among these sub-populations.

## 5. Conclusions

We aimed to investigate the associations of poor sleep quality, depression, anxiety, fatigue, oral pain, and biomarkers of stress and inflammation in newly diagnosed HNC patients. Using network analysis, we found evidence that poor sleep quality, fatigue, CRP, and IL-6 were the most important variables in these complex interconnections. Our findings may assist future studies in disentangling the role of inflammation and psychoneurological symptoms in the progression of HNC-related outcomes over time.

## Figures and Tables

**Table 1:** Characteristics of patients with complete data (the study population) vs. patients with incomplete data.

Characteristics	Patients with Complete Data	Patients with Incomplete Data	Adjusted *p*-Value ^a^
	(*n* = 264)	(*n* = 475)	
Age (mean, SD)	65 (8.2)	62 (10.4)	0.007
Men, No. (%)	209 (79.2%)	340 (71.6%)	0.112
Education level, No. (%) ^b^			
Low	107 (40.5%)	172 (44.8%)	0.560
Middle	77 (29.2%)	94 (24.5%)	
High	80 (30.3%)	118 (30.7%)	
Living together, No. (%) ^b^	215 (81.4%)	270 (70.1%)	0.007
HNC location, No. (%)			
Oral cavity	64 (24.2%)	135 (28.4%)	0.427
Oropharynx	98 (37.1%)	164 (34.5%)	
Hypopharynx	17 (6.4%)	35 (7.4%)	
Larynx	81 (30.7%)	124 (26.1%)	
Unknown primary	4 (1.5%)	17 (3.6%)	
HNC stage, No. (%)			
I	67 (25.4%)	96 (20.2%)	0.559
II	48 (18.2%)	84 (17.7%)	
III	40 (15.2%)	87 (18.3%)	
IV	109 (41.3%)	208 (43.8%)	
ECOG performance status, No. (%)			
0	191 (72.3%)	316 (66.5%)	0.238
1 or more	73 (27.7%)	159 (33.5%)	
Comorbidity, No. (%) ^b^			
None	86 (33.7%)	118 (26.6%)	0.427
Mild	93 (36.5%)	171 (38.5%)	
Moderate	51 (20%)	104 (23.4%)	
Severe	25 (9.8%)	51 (11.5%)	
Excessive alcohol consumption, No. (%) ^b^	58 (22.0%)	71 (22.8%)	0.805
Smoking daily, No. (%) ^b^	57 (21.7%)	70 (22.7%)	0.805
BMI (kg/m^2^) ^b^, mean (SD)	26.1 (4.5)	25.3 (4.6)	0.112
Sleep (PSQI score) ^b^, median (IQR)	5.0 (3.0–7.0)	5.0 (3.0–9.0)	0.600
Depression (HADS-D score) ^b^, median (IQR)	3.0 (1.0–6.0)	3.0 (1.0–6.0)	0.791
Anxiety (HADS-A score) ^b^, median (IQR)	5.0 (3.0–7.8)	5 (3.0–8.0)	0.600
Oral pain (EORTC-H&N35) ^b^, median (IQR)	16.7 (8.3–33.3)	25 (8.3–50.0)	0.112
Fatigue (MFI general fatigue) ^b^, median (IQR)	9.0 (5.0–13.0)	11.5 (6.0–14.0)	0.007
Cortisol slope (nmol/L/hour) ^b^, median (IQR)	0.47 (0.25–0.78)	0.48 (0.28–0.77)	0.791
CRP (mg/L) ^b^, median (IQR)	2.8 (2.5–5.5)	3.1 (2.4–8.0)	0.238
IL-6 (pg/mL) ^b^, median (IQR)	1.03 (0.66–1.72)	1.11 (0.66–1.84)	0.427
IL-10 (pg/mL) ^b^, median (IQR)	0.24 (0.18–0.36)	0.27 (0.19–0.40)	0.222
TNF-α (pg/mL) ^b^, median (IQR)	2.72 (2.32–3.37)	2.79 (2.31–3.44)	0.600

^a^ *p*-values were obtained from comparison statistics: chi-square test for categorical variables, *t*-test for normally distributed continuous variables, or Mann-Whitney U test for non-normally distributed variables. Corrections for multiple comparison were performed with false discovery rates approach. Statistically significant variables (in bold) were defined by *p* value < 0.05. ^b^ There were 91 missing values on education level, 90 on living arrangements, 40 on comorbidity score, 164 on excessive alcohol consumption, 167 on smoking, 169 on BMI, 179 on PSQI score, 142 on oral pain score, 142 on HADS-D score, 144 on HADS-A score, 177 on MFI general fatigue score, 340 on cortisol slope, and 93 on each inflammation marker (CRP, IL-6, IL-10, and TNF-α). Abbreviations: BMI, body mass index; CRP, c-reactive protein; HADS-A, the hospital anxiety and depression scale—anxiety subscale; HADS-D, the hospital anxiety and depression scale—depression subscale; HNC, head and neck cancer; IL, interleukin; IQR, interquartile range; PSQI, Pittsburgh sleep quality index; MFI, multidimensional fatigue inventory; SD, standard deviation; TNF-α, tumor necrosis factor alpha.

**Table 2:** Centrality indices of the network model.

-	Degree	Betweenness	Closeness
Poor sleep quality	5	11.33	0.040
Depression symptoms	4	9.33	0.045
Anxiety symptoms	3	0	0.037
Oral pain	3	3.67	0.042
Fatigue	5	9	0.048
Cortisol slope	1	0	0.028
CRP	3	15.67	0.048
IL-6	4	25.33	0.045
IL-10	2	0	0.033
TNF-α	3	10	0.034
Age	2	8.67	0.043
BMI	1	0	0.026

Anxiety symptoms were measured by the HADS anxiety subscale score; depression symptoms, HADS depression subscale score; fatigue, MFI general fatigue score; oral pain, EORTC QLQ-H&N35 oral pain subscale score; poor sleep quality, PSQI total score. Centrality indices are degree (number of connections), closeness (proximity of a variable to other variables), and betweenness (based on the number of times a variable is located on the shortest path between any pair of other variables). Abbreviations: BMI, body mass index; CRP, c-reactive protein; IL, interleukin; TNF-α, tumor necrosis factor alpha.

## Data Availability

The data analyzed in this article is regulated by the NET-QUBIC project. Any inquiries to access the data can be directed to the NET-QUBIC project. Please refer to NET-QUBIC website (www.kubusproject.nl) detailed information.

## Abbreviations

BMI: body mass index; CRP: c-reactive protein; ECOG: Eastern cooperative oncology group; EORTC QLQ-H&N35: European organization for research and treatment of cancer quality of life questionnaire—HNC-specific module; HADS: hospital anxiety and depression scale; HADS-A: hospital anxiety and depression scale, anxiety subscale; HADS-D: hospital anxiety and depression scale, depression subscale; HNC: head and neck cancer; HPA: hypothalamic-pituitary-adrenal; IL: interleukin; IQR: interquartile range; MFI: multidimensional fatigue inventory; NET-QUBIC: the Netherlands quality of life and biomedical cohort; PSQI: Pittsburgh sleep quality index; SD: standard deviation; TNF-α: tumor necrosis factor alpha.
